# Inhibited Bacterial Adhesion and Biofilm Formation on Quaternized Chitosan-Loaded Titania Nanotubes with Various Diameters

**DOI:** 10.3390/ma9030155

**Published:** 2016-03-03

**Authors:** Wen-tao Lin, Yi-yuan Zhang, Hong-lue Tan, Hai-yong Ao, Zhao-ling Duan, Guo He, Ting-ting Tang

**Affiliations:** 1 Shanghai Key Laboratory of Orthopaedic Implants, Department of Orthopaedic Surgery, Shanghai Ninth People’s Hospital, Shanghai Jiao Tong University School of Medicine, Shanghai 200011, China; wentaolin@126.com (W.-t.L.); hnlc.love@163.com (H.-l.T.); aohyong@126.com (H.-y.A.); 2 State Key Lab of Metal Matrix Composites, School of Materials Science and Engineering, Shanghai Jiao Tong University, Shanghai 200240, China; 07120111@alumni.sjtu.edu.cn; 3 Department of Orthopaedic Surgery, The Second Hospital of Fuzhou Affilated to Xiamen University, Fuzhou 350007, China; zhangyiyuan@medmail.com.cn

**Keywords:** titania nanotubes, quaternised chitosan, bacteria adhesion, biofilm formation, implant-associated infection, antibiotic-resistant staphylococcus

## Abstract

Titania nanotube-based local drug delivery is an attractive strategy for combating implant-associated infection. In our previous study, we demonstrated that the gentamicin-loaded nanotubes could dramatically inhibit bacterial adhesion and biofilm formation on implant surfaces. Considering the overuse of antibiotics may lead to the evolution of antibiotic-resistant bacteria, we synthesized a new quaternized chitosan derivative (hydroxypropyltrimethyl ammonium chloride chitosan, HACC) with a 27% degree of substitution (DS; referred to as 27% HACC) that had a strong antibacterial activity and simultaneously good biocompatibility with osteogenic cells. Titania nanotubes with various diameters (80, 120, 160, and 200 nm) and 200 nm length were loaded with 2 mg of HACC using a lyophilization method and vacuum drying. Two standard strain, methicillin-resistant *Staphylococcus aureus* (American Type Culture Collection 43300) and *Staphylococcus epidermidis* (American Type Culture Collection 35984), and two clinical isolates, *S. aureus* 376 and *S. epidermidis* 389, were selected to investigate the bacterial adhesion at 6 h and biofilm formation at 24, 48, and 72 h on the HACC-loaded nanotubes (NT-H) using the spread plate method, confocal laser scanning microscopy (CLSM), and scanning electron microscopy (SEM). Smooth titanium (Smooth Ti) was also investigated and compared. We found that NT-H could significantly inhibit bacterial adhesion and biofilm formation on its surface compared with Smooth Ti, and the NT-H with 160 nm and 200 nm diameters had stronger antibacterial activity because of the extended HACC release time of NT-H with larger diameters. Therefore, NT-H can significantly improve the antibacterial ability of orthopedic implants and provide a promising strategy to prevent implant-associated infections.

## 1. Introduction

Biomaterial-related infection is one of the most serious postoperative complications of medical implants [1]. Infection rates have been reported to be 1%–4% in primary total joint replacement despite strict sterilization procedures [2]. Systemic antibiotic treatment and local drug delivery are two of the main strategies for the prevention and treatment of implant-associated infections. Compared with systematic administration, local drug delivery is thought to be safer and more effective [3].

Titania nanotubes (TNTs) have attracted much attention due to their low elastic modulus [4] and unique topography [5,6]. Thus, TNTs-based local drug delivery emerged as a promising strategy for preventing implant-associated infections [7,8,9,10,11]. Popat *et al*. [12] have loaded nanotubes with gentamicin and demonstrated that the gentamicin eluted from the nanotubes decreased bacterial adhesion on their surfaces. In our previous study, we investigated the antibacterial ability of the gentamicin-loaded nanotubes (NT-G) with various diameters [13]. We found that NT-G could significantly inhibit bacterial adhesion and biofilm formation on its surface. However, overuse of antibiotics leads to the evolution of antibiotic-resistant bacteria, especially methicillin-resistant *Staphylococcus aureus* (MRSA), the gentamicin-loaded nanotubes are not always successful [14,15,16]. MRSAs are resistant to β-lactam antibiotics (oxacillin, penicillin, and amoxicillin), including third-generation cephalosporins, streptomycin, tetracycline, and sulfonamides; and upon exposure to vancomycin and other glycopeptide antibiotics, certain MRSA strains become less susceptible to these antibiotics [17]. The emergence of resistant strains made implant-associated infections more difficult to treat. In addition, according to previous studies, gentamicin at high local concentrations reduced the viability, proliferation, and alkaline phosphatase activity of the osteoblasts [18,19,20], and inhibited the proliferation and differentiation of human bone marrow mesenchymal stem cells *in vitro* and *in vivo* [21,22], which compromised the bone-healing process. Therefore, it is imperative to investigate a more effective antimicrobial agent against the antibiotic-resistant bacteria that could be loaded into the nanotubes.

Chitosan is a naturally absorbable, non-toxic biopolymer obtained from the exoskeleton of crustaceans [23]. There were much literature demonstrating the remarkable biocompatibility and antibacterial activity of chitosan [24,25,26]. The anti-infective ability of chitosan is due to the electrostatic effect, between −NH^3+^ groups in CS and phosphoryl groups of phospholipid components in bacterial cell walls [27]. However, the antibacterial activity of chitosan is limited by its poor solubility in water at neutral or high pH, except in acidic conditions [23]. Compared to chitosan, interestingly, chitosan derivatives by chemical modification exhibit a higher water solubility and antibacterial activity over all pH ranges [25]. In our previous study, we successfully synthesized a new water-soluble chitosan derivative (hydroxypropyltrimethyl ammonium chloride chitosan, HACC) by using glycidyl trimethylammonium chloride (GTMAC) as a quaternizing agent to react with the nucleophilic amino groups of chitosan [28]. We found that the antibacterial activity and biocompatibility of HACC could be balanced by varying the DS (degree of substitution) of the quaternary ammonium. The antimicrobial activity of water-soluble HACC against bacteria was enhanced upon increasing the DS, but the high DS was cytotoxic and interfered with the proliferation and osteogenic differentiation of human bone marrow-derived mesenchymal stem cells (hMSCs). Thus, HACC, with moderate degrees of substitution, had enhanced antibacterial ability and no cytotoxicity. Our previous report further demonstrated that HACC with moderate degrees of substitution could significantly prevent bacterial adhesion and biofilm formation, including antibiotic-resistant strains (MRSA and MRSE), and downregulates the expression of MecA (methicillin resistance determinant A), which encodes membrane-bound enzymes known to be penicillin-binding proteins [29].

A promising application of HACC is loading it into the nanotubes to deliver a proper dose of antibacterial agent directly to implant-tissue interface to kill planktonic bacteria and to prevent bacterial adhesion and biofilm formation. In this study we investigated the antibacterial ability of various diameter nanotubes loaded with HACC (NT-H), especially the ability to inhibit biofilm formation, and HACC with moderate degrees of substitution (27%) was used. *Staphylococcus aureus*, Methicillin-resistant *Staphylococcus aureus*, and *Staphylococcus epidermidis* contribute to 66% of disastrous orthopedic implant-related infections [30]. Therefore, the standard strains methicillin-resistant *S. aureus* (American Type Culture Collection [ATCC] 43300), *S. epider*midis (ATCC35984), and two clinical isolates, *S. aureus* 376 and *S. epidermidis* 389, were selected in this work, and the four bacterial strains had biofilm-forming properties [29,31].

## 2. Materials and Methods

### 2.1. Materials

TNTs specimens with various diameters (80, 120, 160, and 200 nm) and 200 nm length were prepared *via*electrochemical anodization as described in our previous study [13]. HACC with a degree of substitution of quaternary ammonium of 27% (referred to as 27% HACC) was prepared by combining chitosan and glycidyl trimethylammonium chloride (GTMAC) as previously reported [28,29]. Chitosan (CS) of a molecular weight of 2.0 × 105, and with a N-deacetylation rate of 91.83%, was purchased from Zhejiang Yuhuan Ocean Biochemistry Co. Ltd. (Taizhou, China). Smooth titanium (Smooth Ti) discs were used as a control in all experiments. Both sides of the specimens were sterilized by ultraviolet irradiation before conducting the antibacterial experiments.

### 2.2. Loading of HACC

27% HACC was loaded into the nanotubes by a lyophilization method and vacuum drying [12,32,33]. In brief, the 27% HACC solution of 20 mg/mL was prepared in deionized water. The surfaces of the TNTs were cleaned with deionized water before HACC loading. Then, 10 μL of HACC solution was pipetted onto the nanotubes surfaces and gently spread to ensure even coverage. The specimens were then allowed to dry under vacuum at −45 °C for 2 h. After drying, the loading step was repeated until the nanotubes were loaded with 2 mg of HACC. After the final drying step, the surfaces were quickly rinsed by pipetting 1 ml of phosphate-buffered saline (PBS) over the surface to remove any excess HACC. The rinse solutions were collected and stored for further analysis.

### 2.3. Characterization of Drug Release from TNTs

The release kinetics of 27% HACC from the nanotubes was determined by the anthrone sulfuric acid reaction [29,34,35]. Three discs of NT-H with different diameters were each individually immersed in 1 ml of PBS in a 48-well plate (Costar3548, Corning Incorporated, Corning, NY, USA) at a temperature of 37 °C and agitated at 100 rpm. No HACC-loaded nanotubes immersed in 1 mL of PBS were used as a parallel control. The solution was replaced with 1 mL of fresh PBS every time samples were collected. All the samples were taken after specific intervals to determine the release kinetics and collected periodically for up to 60 h. The samples were analyzed for HACC content using the anthrone sulfuric acid reaction. This method was used to measure the total carbohydrates (which are derived from the degradation of HACC) in the eluate from nanotubes because the chemical structure of HACC is a copolymer of *b*-(1-4)-d-glucosamine and N-acetyl-*b*-(1-4)-d-glucosamine. The degradation products of the copolymer, namely N-acetyl glucosamine molecules, undergo both the hydrolysis of glycosidic bonds and the dehydration of monomers to produce furfuraldehyde derivatives under heat and a strongly acidic environment. These compounds react with anthrone and produce colored products, which exhibit maximum absorbance at a wavelength of 630 nm [29]. Briefly, 40 μL standard samples (12.5, 18.75, 37.5, 50, 75, 100, 150, 200, 300, 400, 600, 800, 1200, 1600, and 2400 mg/mL glucose), 40 μL PBS taken from NT-H and a 40 μL sample taken from no HACC-loaded nanotubes preparations were placed into individual 96-well flat-bottom plates. These plates were vortexed gently and were incubated at 4 °C for 15 min. Next, 0.1 mL freshly prepared anthrone solution was added to each well using a micropipette, and the plate was vortexed gently but thoroughly and incubated at 92 °C in a non-shaking water bath. After three minutes, the plate was transferred to a non-shaking water bath at room temperature for 5 min to stop the reaction. Absorbance was determined using a Synergy HT multidetection microplate reader at 630 nm. A standard curve with known concentrations of the glucose standard samples was used to determine the total carbohydrate concentration. The amount of carbohydrate was measured over a period of 60 h.

### 2.4. Preparation and Characterization of Bacteria

Methicillin-resistant *Staphylococcus aureus* (ATCC43300) was purchased in a freeze-dried form from the American Type Culture Collection (Manassas, VA, USA). *S. epidermidis* (ATCC35984) was kindly provided by Di Qu (Laboratory of Medical Molecular Virology, Shanghai Medical College, Fudan University, Shanghai, China). The clinical isolate *S. aureus* 376 and *S. epidermidis* 389 were kindly provided by Saïd Jabbouri (Université du Littoral Côte D’Opale, Boulognesur-Mer, France). Our previous studies demonstrated that the four tested strains were biofilm-producing bacterial strains [31,36]. These strains were stored at −80 °C as glycerol stocks. Before bacterial inoculation, the strains were propagated overnight on tryptone soy agar (TSA) medium at 37 °C. A sterile 10 μL loop was used to withdraw bacteria colonies from the TSA, which were then inoculated into 10 mL of BBL^TM^ Trypticase^TM^ soy broth (TSB, BD Biosciences, Franklin Lakes, NJ, USA) and cultured for approximately 16 h on a shaker at 250 rpm and 37 °C. Cells were then harvested by centrifugation (8000× g for 10 min). The minimum inhibiting concentrations (MICs) of HACC against methicillin-resistant *S. aureus* (ATCC 43300), *S. epidermidis* (ATCC 35984), *S. aureus* 376, and *S. epidermidis* 389 were determined by a microtiter broth dilution method as previously described [31,37,38].

### 2.5. Bacterial Adhesion Assay Using the Spread Plate Method

Bacterial adhesion on Smooth Ti and NT-H with different diameters was investigated by the spread plate method, which has been described elsewhere [39,40]. The inocula of the four strains were re-suspended to a final density of 1 × 10^6^ colony forming units (CFUs)/mL in TSB using McFarland standards. The Smooth Ti and different-diameter NT-H disks was incubated with 1 mL of the suspension in a 48-well plate at 37 °C with agitation at 100 rpm for 6 h. Then, the specimens were removed with sterile forceps, placed into a fresh 48-well plate and gently washed with sterile PBS three times to remove loosely-adherent bacteria. The specimens were then placed in 0.5 mL of TSB, and the adherent bacteria on the discs were dislodged by ultrasonication (5 min) in a 150 W ultrasonic bath (B3500S-MT, Branson Ultrasonics Co., Shanghai, China) operating at a frequency of 50 Hz. The ultrasonication was followed by rapid vortex mixing (Vortex Genie 2, Scientific Industries, Bohemia, NY, USA) at maximum power for 1 min to remove bacteria that had adhered to the surfaces. This method is known to be effective for removing biomaterial-adherent bacteria [41,42]. The vortexed solutions were plated in triplicate onto TSA and then incubated at 37 °C for 24 h. The number of CFUs on the TSA was counted, the amount of bacteria adhesion on the substrates was calculated, and is expressed relative to the surface area of the sample (CFUs/mm^2^).

### 2.6. Biofilm Formation Assay Using the Tissue Culture Plate (TCP) Method

The TCP assay method has been considered the standard test to detect biofilm formation [43,44]. In brief, the specimens were incubated with bacterial suspensions of 1 × 10^6^ CFUs/mL in TSB for 24, 48, and 72 h. Another 48-well plate containing the specimens and TSB was used as the negative control. The samples were gently washed with PBS three times to remove free-floating planktonic bacteria. The biofilms formed on the specimens were dried at 60 °C for 1 h and stained with 200 μL of a 0.1% (wt/vol) aqueous solution of crystal violet (CV) at room temperature for 5 min. The samples were rinsed twice with deionized water to remove excess stain. After drying at 37 °C for 2 h, the biofilm formation on the surfaces was quantified by solubilization of the CV stain in 200 μL of 30% (wt/vol) glacial acetic acid for 10 min with agitation at 300 rpm. The CV concentration was determined at a wavelength of 492 nm [45]. The mean absorbance obtained from the medium control well was deducted from the test absorbance values.

### 2.7. Observation of Bacterial Adhesion and Biofilm Formation Using Scanning Electron Microscopy (SEM)

The specimens were incubated with bacterial suspensions of 1 × 10^6^ CFUs/mL in TSB for 6, 24, 48, and 72 h. At the end of the incubation period, the substrates were gently washed three times with PBS. Then, the surfaces were fixed in 2.5% glutaraldehyde for 2 h at 4 °C, followed by washing three times with cacodylate buffer and dehydrated in serial concentrations of ethanol (25%, 50%, 75%, 95%, and 100%) for 10 min each. Then, the surfaces were dried with hexamethyldisilazane (HMDS) (Polysciences) for 10 min. The HMDS was removed, and the surfaces were air dried for 30 min. The samples were subsequently dried with a critical point dryer, sputter coated with gold, and observed using a scanning electron microscope (Joel JSM-6310LV, JEOL Ltd., Tokyo, Japan).

### 2.8 Observation of Bacterial Adhesion and Biofilms Formation Using Confocal Laser Scanning Microscopy (CLSM)

The bacterial adhesion and biofilms formation on Smooth Ti and four groups of NT-H were also observed using CLSM. Similarly, the specimens were removed at four different time points and followed by gently washed three times with PBS. The samples were stained with 300 μL of combination dye (LIVE/DEAD BacLight bacteria viability kits, Molecular Probes, L13152) for 15 min in the dark and then were analyzed with a confocal laser scanning microscope (Leica TCS SP2; Leica Microsystems, Heidelberg, Germany). The viable bacteria with intact cell membranes appear fluorescent green, whereas nonviable bacteria with damaged membranes appear fluorescent red. The images were acquired from random positions on the surfaces of the samples. We used ATCC 43300 for the CLSM and SEM observation assays in this study.

### 2.9 Statistical Analysis

All experiments were conducted in triplicate and repeated three times. The results are expressed as mean ± standard deviation. The one-way analysis of variance and least significant difference (LSD) *post hoc* tests were used to determine the level of significance; *P* < 0.05 was defined as significant and *P* < 0.01 was defined as highly significant. The statistical analyses were performed using SPSS software version 13.0.1 (SPSS Inc., Chicago, IL, USA).

## 3. Results

### 3.1. Loading Efficiency of HACC in Different Diameter Nanotubes

**Figure 1:**
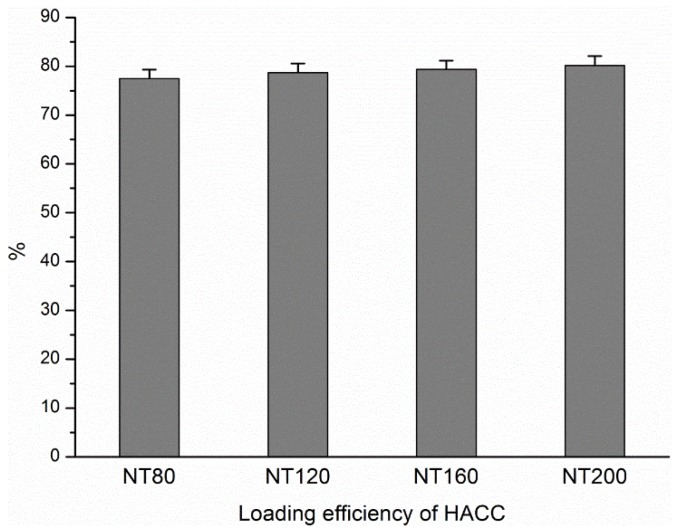
Loading efficiency of HACC in nanotubes with different diameters.

As described above, HACC was loaded into nanotubes using a lyophilization method and vacuum drying in this study, and the nanotubes were filled with 2 mg of HACC. The loading efficiency of HACC in the nanotubes was evaluated prior to the HACC release assay. The concentrations of the rinse solutions were measured by the previously described anthrone sulfuric acid reaction. The loading efficiency was calculated from the formula η = (*m_o_* – *m_r_*)/*m_o_*, where η is the loading efficiency, *m_o_* is the amount of HACC loaded in the nanotubes (2 mg), and *m_r_* is the amount of HACC in the rinse solution. Figure 1 shows the loading efficiencies of different diameter NT-H. The results indicate that approximately 75%–80% of HACC is retained in the nanotubes after the initial wash. No statistically significant difference was found among the four different diameter NT-H (*P* > 0.05).

### 3.2. HACC Release from the Nanotubes

**Figure 2:**
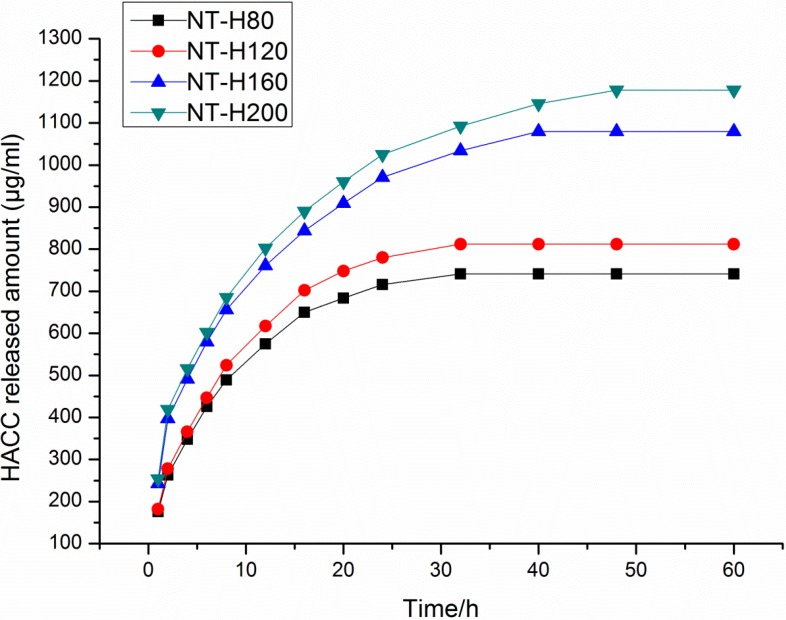
Cumulative HACC release profiles from HACC loaded nanotubes with different diameters, expressed in µg/mL.

Figure 2 shows that HACC release from different diameter nanotubes, which is expressed in µg/mL. There is higher sustained HACC release from the nanotubes with larger diameters (160 nm or 200 nm) than from those with smaller diameters (80 nm or 120 nm). The majority of HACC was released from NT-H160 and NT-H200 after approximately 24 h and from NT-H80 and NT-H120 after approximately 12 h. We observed that HACC release from NT-H could be divided into two parts: initial burst release and relatively slow release. After a high initial release, the amount of HACC eluted from the nanotubes remained nearly constant. As shown in Table 1, the total release of HACC from NT-H80, NT-H120, NT-H160, and NT-H200 was 741, 812, 1080, and 1178 µg, respectively.

### 3.3. The MICs of the Bacterial Strains

As shown in Table 2, the MICs of HACC were 64 µg/mL, 32 µg/mL, 32 µg/mL, and 32 µg/mL against ATCC 43300, ATCC 35984, *S. aureus* 376, and *S. epidermidis* 389, respectively. It indicated that the four tested strains were susceptible to HACC.

### 3.4. Inhibition of Bacterial Adhesion and Biofilm Formation on the NT-H

**Figure 3:**
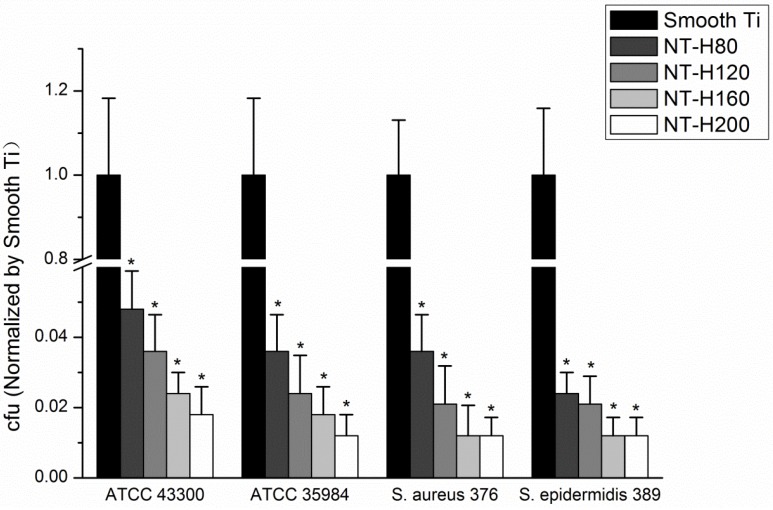
The number of viable bacteria adhered on Smooth Ti and NT-H surfaces at 6 h. The number of viable bacteria was counted and normalized to the counts from the Smooth Ti control for each bacterial strain. * denotes a significant difference compared to Smooth Ti (*P* < 0.01).

**Figure 4:**
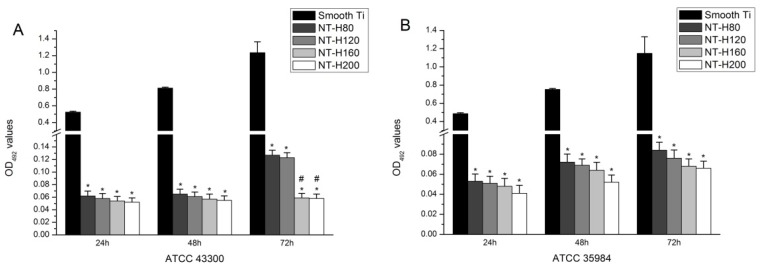

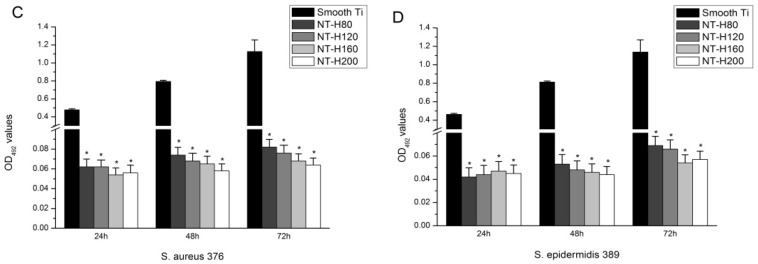
Biofilm formation of the three bacterial strains. (**A**) ATCC 43300; (**B**) ATCC 35984; (**C**) *Staphylococcus aureus* 376; and (**D**) *S. epidermidis* 389 on Smooth Ti and NT-H surfaces at 24 h, 48 h, and 72 h, as detected by the tissue culture plate method. * denotes a significant difference compared to Smooth Ti (*P* < 0.01). # denotes a significant difference compared to NT-H80 and NT-H120 (*P* < 0.01).

The number of viable bacteria that adhered to NT-H at the 6 h time point was determined by the spread-plate method. As shown in Figure 3, the number of living bacteria of the four tested strains on the surface of the NT-H was significantly lower than on the Smooth Ti (*P* < 0.01), and no difference was observed among NT-H80, NT-H120, NT-H160, and NT-H200 (*P* > 0.05). The biofilm formation of the four tested strains on the specimens was assessed by crystal-violet staining using the TCP method. As shown in Figure 4, it can be observed that the A_492_ values of the four tested strains on NT-H were significantly lower than on Smooth Ti at the 24, 48, and 72 h time points (*P* < 0.01) which indicates almost no biofilm formation on NT-H surfaces. For the A_492_ values of ATCC 35984, *S. aureus* 376, and *S. epidermidis* 389, similar to the bacterial adhesion results from the spread plate assay, no difference can be observed among NT-H samples with different diameters at the three time points (*P* > 0.05), while the A_492_ values of ATCC 43300 on NT-H160 and NT-H200 were lower than on NT-H80 and NT-H120 at the 72 h time point (*P* < 0.01).

### 3.5. SEM and CLSM Observation

The bacterial adhesion and biofilm formation on the surfaces of Smooth Ti and NT-H were observed using SEM and CLSM at the 6, 24, 48, and 72 h time points. We selected the ATCC 43300 for SEM and CLSM observation in this study.

**Figure 5:**
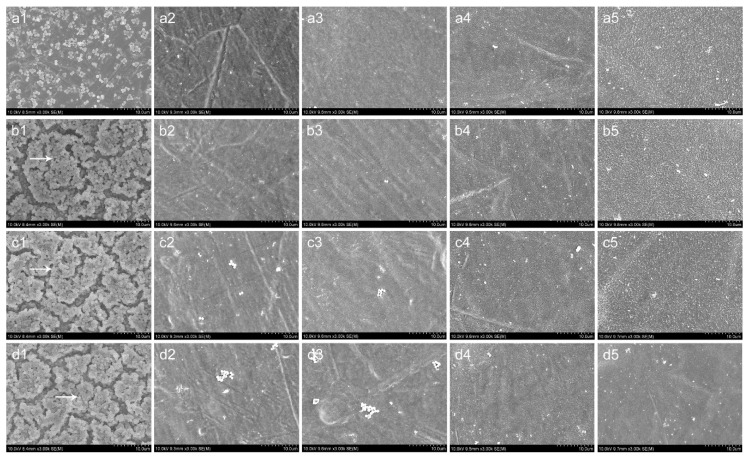
SEM images of ATCC 43300 adhesion and biofilm formation on different surfaces. (1) Smooth Ti; (2) NT-H80; (3) NT-H120; (4) NT-H160; (5) NT-H200 after (**a**) 6 h; (**b**) 24 h; (**c**) 48 h; and (**d**) 72 h incubation. Arrow head indicates the bacteria colony in biofilm. The magnification level is ×3,000. The scale bar is 10 µm.

**Figure 6:**
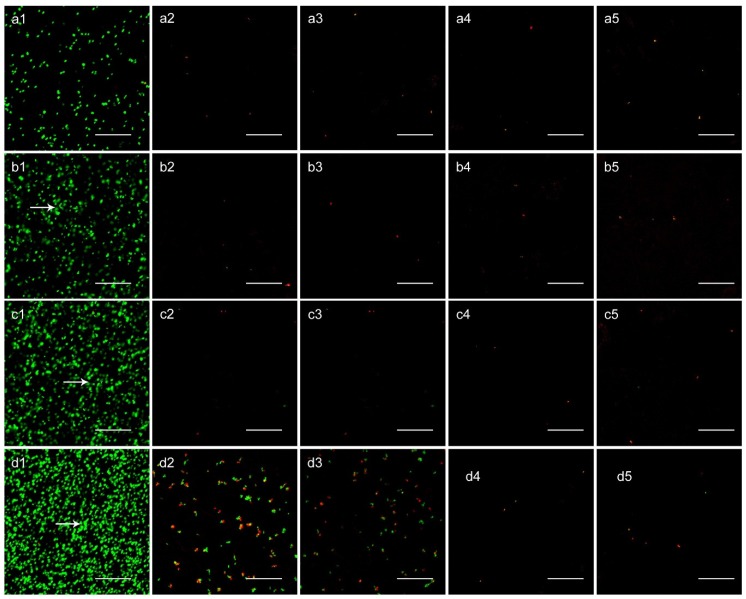
CLSM analysis of bacterial viability on different surfaces. (1) Smooth Ti; (2) NT-H80; (3) NT-H120; (4) NT-H160; (5) NT-H200 incubated with ATCC 43300 for (**a**) 6 h; (**b**) 24 h; (**c**) 48 h; and (**d**) 72 h. Arrow head indicates the bacteria colony in biofilm. The magnification level is ×400. The scale bar is 50 µm.

As shown in Figure 5, there were a few single bacterial colonies scattered on the surfaces of NT-H80 and NT-H120 at the 6, 24, and 48 h time points (a2, b2, c2, a3, b3, c3) and on the surfaces of NT-H160 and NT-H200 at the four time points (a4, b4, c4, d4, a5, b5, c5, d5). The colonies on the NT-H80 and NT-H120 surfaces were more obvious than those on NT-H160 and NT-H200 at the 72 h time point. In contrast, it could be observed that many multiple bacterial colonies adhered to the surfaces of the Smooth Ti at the 6 h time point (a1) and formed biofilm with a multilayer structure at the 24, 48, and 72 h time points (b1, c1, d1). In Figure 6, we could observe that an intense fluorescence on the surface of the Smooth Ti at the 24, 48, and 72 h time points (b1, c1, d1), which indicated that significant biofilm formation. The bacterial colonies were sparsely distributed on the surface of NT-H at different time points, which indicates no biofilm formation. Similar to the results of SEM observation, there were fewer bacterial colonies on the surfaces of NT-H160 and NT-H200 compared to those of NT-H80 and NT120 at the 72 h time point (d2, d3, d4, d5).

## 4. Discussion

Our previous reports demonstrated that HACC with moderate degrees of substitution could significantly inhibit biofilm formation without cytotoxicity, and dramatically downregulated the expression of icaAD, which encodes essential enzymes for polysaccharide intercellular adhesion (PIA) biosynthesis, both in new biofilms and in preexisting biofilms, upregulated the expression level of icaR, which negatively mediates icaAD expression [29,31]. The exact mechanisms of the antibacterial activities of quaternized chitosan are still unknown. The prerequisite for antibacterial activity of quaternized chitosan is its polycationic structure. Electrostatic interaction between the polycationic structure and the predominantly anionic components of the microorganisms play a fundamental role in antibacterial activity. The quaternized chitosan could form a film around the cell that protects cells against nutrient transport through the microbial cell membrane [46], or interact with the cell surface to alter cell permeability. The increased membrane permeability leads to destabilization of the cell membrane, leakage of intracellular substances and, ultimately, the death of cells [25,47]. We fabricated TNTs with various diameters using electrochemical anodization in our previous study, and we filled nanotubes with 27% HACC using a lyophilization method and vacuum drying to achieve local delivery in this study.

Once the biofilm forms, it is extremely difficult to eliminate [48,49]. It is postulated that biofilms contribute to antibiotic resistance by at least three mechanisms: reduced antibiotic penetration across the extracellular polymeric substance, a favorable environment within the inner layers, and bacteria cell differentiation and role specialization providing increased protection [50]. Pathogenic bacteria are capable of persisting in a biofilm in the presence of antibiotics at levels that are 1000-fold higher than those necessary to eradicate a planktonic population [51].

The initial adhesion of bacteria to biomaterial surfaces is the critical event for the pathogenesis of foreign body infections [52]. Reducing bacterial adhesion during the initial 6 h period following implantation is particularly important to avoid implant-associated infection [53]. It has been suggested that if the antibiotics or other antibacterial agents are able to prevent initial bacterial adherence to surfaces, the subsequent step of biofilm formation would also be inhibited [54]. Therefore, an effective measure to combat the implant-associated infection is to inhibit bacterial adhesion prior to biofilm formation. We can observe in Table 1 that an initial burst of release in all four groups of NT-H with different diameters. The high initial concentrations of released HACC would efficiently kill planktonic bacteria to inhibit bacterial adhesion on the nanotubes surface and the total amounts of HACC released from NT-H80, NT-H120, NT-H160, and NT-H200 within 60 h were 741, 812, 1080, and 1178 µg, respectively, which were all above the MICs of each group of tested strains (64 µg/mL for ATCC 43300, 32 µg/mL for ATCC 35984, 32 µg/mL for *S. aureus* 376, and 32 µg/mL for *S. epidermidis* 389). As described previously, the spread plate method, tissue culture plate, CLSM, and SEM were used to investigate the ability of NT-H with different diameters to prevent bacterial adhesion and biofilm formation for four tested strains, ATCC 43300, ATCC 35984, *S. aureus* 376, and *S. epidermidis* 389. Our results demonstrated that for all four tested strains, including MRSA, all groups of NT-H could significantly inhibit the bacterial adhesion, and there was no biofilm formation on the NT-H surfaces at different time points. Compared with the NT-H, there were far more bacteria adhered to the surface of Smooth Ti at the 6 h time point and formed obvious biofilm at the 24, 48, and 72 h time points for all tested strains.

Additionally, we found that for ATCC 43300, the numbers of viable colonies on the NT-H with 160 nm and 200 nm diameters were significantly lower than those on NT-H with 80 nm and 120 nm diameters at the 72 h time point. Similar to NT-G, these results indicated that the larger the diameters of the NT were, the stronger the antibacterial activity would be, because of the extended HACC release time of the NT with larger diameters. In addition, our previous results demonstrated that the majority of gentamicin was released from NT-G80 and NT-G120 after approximately 9 h and from NT-G160 and NT-G200 after approximately 21 h [15]. In this study, our results showed that the initial HACC release time of the smaller diameter nanotubes (NT-H80 and NT-H120) was extended to approximately 12 h, and that of the larger diameter nanotubes (NT-H160 and NT-H200) was extended to approximately 24 h. It indicated that NT-H had a higher sustained HACC release comparing to NT-G with the same diameter. The more sustainable HACC release from nanotubes may be related to its larger molecular size compared with the gentamicin.

In this study, we did not set the control of nanotubes without any drug loading; however, the antibacterial potential of nano-surfaces have been well studied in previous publications. Colon *et al*. [55] compared the behavior of *S. epidermidis* on nanostructured and microstructured ZnO and TiO_2_. They found that *S. epidermidis* adhesion on nanostructured ZnO and TiO_2_ was less than that observed on the microphase formulations. Puckett *et al.* [56] found that compared to conventional (nanosmooth) titanium, the nanorough titanium surfaces decreased the bacteria adherence, while nanotubular and nanotextured titanium resulted in an increase of bacterial attachment. In our previous research, we found that the initial adhesion and colonization of *S. epidermidis* on the surfaces of nanotubes were significantly reduced compared with the control samples, especially on the 80 nm nanotubes [57]. In addition, our another report also demonstrated that the unloaded nanotubes with four different diameters have moderate antibacterial activity, and the nanotubes with 80 nm or 120 nm diameters exhibited better antibacterial ability compared those with 160 nm or 200 nm diameters [15].

## 5. Conclusions

This *in vitro* study demonstrated that 27% HACC-loaded nanotubes could dramatically inhibit surface biofilm formation by *staphylococci*, including antibiotic-resistant *staphylococci*. Further studies showed that the larger the diameters of the nanotubes were, the stronger the antibacterial activity would be. This study thus provides a promising strategy to the orthopedic field on methods to inhibit bacterial biofilm formation on implant surface and prevent implant-related infections.

## Figures and Tables

**Table 1:** The initial and total release of HACC in 60 h.

Specimen	Total HACC Loaded (μg ^a^/one disc)	Initial Release (μg ^b^)	Total Release (μg)
NT-H80	1549	575	741
NT-H120	1573	617	812
NT-H160	1587	971	1080
NT-H200	1603	1025	1178

Notes: ^a^ The amount of HACC retained in the nanotubes after the initial wash: 2 mg × η (the loading efficiency). ^b^ The amount of HACC released in the initial high-release period before the rate of HACC release became approximately constant.

**Table 2:** The MICs of the four tested strains.

Microorganism	MIC (μg/mL)
ATCC43300	64
ATCC35984	32
*S. aureus* 376	32
*S. epidermidis* 389	32
